# Age differences in adaptation of medial-lateral gait parameters during split-belt treadmill walking

**DOI:** 10.1038/s41598-021-00515-z

**Published:** 2021-10-27

**Authors:** Tyler Fettrow, Kathleen Hupfeld, Hendrik Reimann, Julia Choi, Chris Hass, Rachael Seidler

**Affiliations:** 1 Department of Applied Physiology and Kinesiology, University of Florida, Gainesville, FL 32605 USA; 2 Department of Kinesiology and Applied Physiology, University of Delaware, Newark, DE 19713 USA

**Keywords:** Control theory, Ageing, Dynamical systems, Bioenergetics

## Abstract

The split-belt treadmill has been used to examine the adaptation of spatial and temporal gait parameters. Historically, similar studies have focused on anterior-posterior (AP) spatiotemporal gait parameters because this paradigm is primarily a perturbation in the AP direction, but it is important to understand whether and how medial-lateral (ML) control adapts in this scenario. The ML control of balance must be actively controlled and adapted in different walking environments. Furthermore, it is well established that older adults have balance difficulties. Therefore, we seek to determine whether ML balance adaptation differs in older age. We analyzed split belt induced changes in gait parameters including variables which inform us about ML balance control in younger and older adults. Our primary finding is that younger adults showed sustained asymmetric changes in these ML balance parameters during the split condition. Specifically, younger adults sustained a greater displacement between their fast stance foot and their upper body, relative to the slow stance foot, in the ML direction. This finding suggests that younger adults may be exploiting passive dynamics in the ML direction, which may be more metabolically efficient. Older adults did not display the same degree of asymmetry, suggesting older adults may be more concerned about maintaining a stable gait.

## Introduction

The upright human body is biomechanically unstable and requires active control by the nervous system to stabilize. We do not have a comprehensive understanding of how humans control balance during walking, or how this control changes with age. The ability to dynamically adapt locomotion is critical for maintaining balance. In the event that one slips or trips, a quick adjustment must be made in order to maintain balance^1,2^. New locomotor patterns must also be adopted to safely navigate along a narrow path^3^ or on uneven terrain^4^. Furthermore, we know older adults struggle with the ability to maintain balance^5^ and have difficulties adapting new motor patterns^6^. Here we investigate whether older and younger adults adapt differently to a split belt treadmill paradigm, which comprises walking on two belts moving at differing speeds. Our particular emphasis is on the medial-lateral (ML) control of balance.

The split belt treadmill paradigm has been used extensively over the past two decades to study locomotor adaptation. This paradigm uses two treadmill belts, one for each leg, that move at different speeds, causing an initial asymmetry in the gait pattern. Over multiple steps, people adapt to a new pattern of walking. The observed adaptation during split belt treadmill walking is commonly quantified as changes in the spatiotemporal gait parameters (step velocity, step time, and step position) to make the gait cycle more symmetric^7^. Prokop et al initially stated that adaptation to split belt treadmill walking occurs within 1–3 strides^8^. However, more recent research suggests > 100 strides are necessary to reach a plateau in the commonly quantified spatiotemporal gait parameters^9^. The discrepancy in reports of adaptation rate could be linked to varying methods used to determine rate of adaptation, as well as which gait parameters are assessed. Mounting evidence points to differences in adaptation between intralimb variables (stance and swing time) and interlimb variables (double support time and step length), where the intralimb variables adapt more quickly^10^. Historically, split belt treadmill analyses have focused on step length, step time, stance time, and associated joint angle changes (e.g.^11–13^), whereas ML changes to balance during walking have only been assessed during split belt treadmill adaptation within the past few years (e.g.^14–16^).

The balance problem is generally understood to require more active control in the ML direction^17–19^, implied by modeling and experimental work showing differences in the source of the variability of foot placement between the anterior-posterior (AP) and ML directions. This supports a growing consensus that step length and cadence are separately controlled from ML gait parameters such as ankle roll (active modulation of center of pressure (CoP) under the stance foot) and step width. But the step timing and duration of the single and double stance periods also affect balance. These timing parameters are shared between the AP and ML directions, so the AP changes enforced by the split belt treadmill paradigm represent a challenge to balance in the ML direction. We hypothesize that gait parameters specific to ML balance control are also modulated throughout the split belt treadmill protocol, but at different rates than the commonly assessed spatiotemporal variables.

Roper et al found modifications of ML ground reaction forces during adaptation in healthy young adults^14^, but these modifications seemed to coincide with the ground reaction forces dictated by the speed of the foot; thus it is not clear whether these changes were adaptive or simply reflect gait speed changes. Buurke et al sought to investigate whether ML gait parameters, including the margin of stability, are altered in the split belt treadmill paradigm^15^. Margin of stability is a measure of dynamic stability that takes into account the center of mass (CoM) position and velocity relative to the base of support (CoP)^20^. The authors found that the CoP and the margin of stability are changed over many steps when participants are adjusting to the *split* condition, but these variables did not exhibit classic aftereffects (i.e. a performance change when the belts are returned to the same speeds that is in the opposite direction of what occurred during the *split* condition), indicating that these changes may have been more strategic rather than adaptive per se. Instead, the margin of stability appeared to be closely related to the amount of time spent in stance^21^, at least in young healthy adults.

To our knowledge, only one other study has investigated ML gait parameter adaptations in split belt treadmill in older adults. Vervoort et al found larger ML margin of stability in older adults, compared to younger adults, throughout the adaptation portion of the split belt treadmill paradigm, suggesting that older adults are concerned more about balance^16^. Here we expand on this work by investigating whether ML balance control is adapted during split belt treadmill walking. We test for changes in ML gait parameters during split belt treadmill adaptation, and we also examine whether there are aftereffects during subsequent tied-belt walking. We define discrete variables for seven different gait parameters and calculate the number of steps to plateau and the magnitude of the changes for each parameter to answer two distinct questions: (1) Are there age differences in the plateau and magnitude of gait parameter changes during *split* walking? (2) Do the gait parameters that are more associated with the control of balance exhibit differences in steps to plateau during the *split* condition and in their magnitude of aftereffects, compared to traditional parameters, reflecting separate neural control?

## Methods

### Participants

Thirty-seven young adults (19 female, $22.4 \pm 3.78$ years) and 28 older adults (12 female, $72.5 \pm 5.26$ years) participated in this study. Handrails were installed on the sides of the treadmill to prevent falls, if necessary, while performing the experiment. No harness was used for this experiment. Due to force plate data corruption and/or marker occlusions we excluded six younger and seven older adults’ data from analysis, leaving 31 younger adults and 21 older adults. This experiment was approved by the University of Florida Institutional Review Board. All participants provided written informed consent to participate.

Exclusion criteria included: History of any neurologic condition, e.g., stroke, Parkinson’s disease, seizures, or a concussion in the last six months. Psychiatric condition, e.g., active depression or bipolar disorder. As part of the larger study, subjects were also screened for magnetic resonance imaging (MRI) and transcranial magnetic stimulation eligibility^22^. We also excluded those who self-reported smoking, consuming more than two alcoholic drinks per day on average or a history of treatment for alcoholism, as participants also underwent GABA MR spectroscopy. We excluded those with any contraindications for these devices, e.g., implanted metal, claustrophobia, or pregnancy. We also excluded individuals who reported taking medications which are contraindications for these devices within the previous 30 days^23^. Such medications included those commonly used to treat psychiatric conditions e.g., depression, bipolar disorder, anxiety, attention-deficit/hyperactivity disorder, and schizophrenia, pain, and seizures, as well as some antiviral and chemotherapy medications.

Prior to enrollment, we screened participants for suspected cognitive impairment over the phone using the Telephone Interview for Cognitive Status^24^. We excluded those who scored < 21 of 39 points; this is equivalent to scoring < 25 points on the Mini-Mental State Exam (MMSE) and indicates probable cognitive impairment. At the first testing session, participants were re-screened for cognitive impairment using the Montreal Cognitive Assessment^25^; we excluded those who scored < 23 of 30 points^26^. None of the subjects had walked on a split-belt treadmill prior to participation in this study.

Participants walked on an instrumented split-belt treadmill (Bertec Inc., Columbus, Ohio, USA) with embedded force plates that captured kinetic data at 1200 Hz. Sixteen passive reflective markers were placed according to the Vicon Plug-in-Gait (Vicon, Oxford, UK) lower body marker system^27^. Kinematic data were collected at 120 Hz using 8 cameras surrounding the treadmill. Of those participants that are included in this analysis, kinematic data were collected with a Vicon Nexus (Vicon, Oxford, UK) motion capture system for 37 participants (22 young and 15 old) and a Qualisys Track Manager (Qualisys, Göteborg, Sweden) motion capture system for 15 participants (9 young and 6 old) due to construction.

### Split-belt adaptation assessments

We first administered a treadmill warm-up lasting 5 minutes at participants’ self-selected speed to allow them to accommodate to walking on the treadmill. For the remainder of the walking trials, the belts moved at a fixed speed for all participants. We then stopped the treadmill before beginning the baseline walking trials, consisting of a *baseline slow* (0.7 m s^−1^), *baseline fast* (1.4 m s^−1^), and *baseline slow* (0.7 m s^−1^) walking trial, each lasting two minutes. These trials acclimated the participants to the slow and fast walking speeds. Then we began the *split* trial, where the left belt was moving at the fast speed, and the right belt was moving at the slow speed, lasting 10 minutes^28^. The *split* trial was followed by an *after* adaptation trial, where both belts were fixed at the slow speed to identify any aftereffects. Participants also performed a *readaptation* and a *washout* trial. For this analysis, we did not include the second *baseline slow*, *readaptation*, or *washout* trials. Table 1 provides the condition names, durations, and belt speeds for each trial. The green box indicates the trials used in the present analyses. For all trials, the treadmill acceleration was set to 0.5 m s^−2^.

**Table 1:**
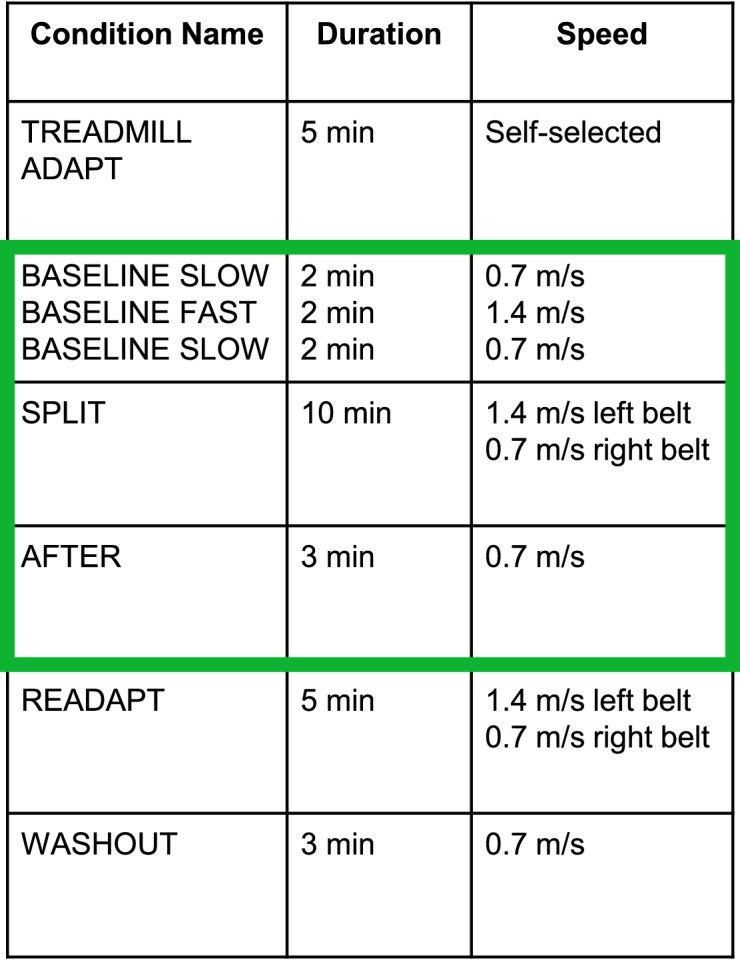
Order of walking protocols for the split-belt adaptation paradigm.

For this analysis, we only focus on the highlighted (green) sections.

### Data management and organization

Kinematic data were low pass filtered with a 4th order Butterworth filter at a cut-off frequency of 10 Hz. Small gaps in the marker data of up to 100 ms length from occlusions were filled using cubic splines. Time points with occlusions greater than 100 ms were excluded from further analysis. Due to limited marker supply, we used the middle of the posterior hip markers as a proxy for the body CoM position^29^. We refer to this as the center of mass (CoM) throughout the manuscript.

We identified heel-strikes as the local maxima of forward progression of the heel marker, and push-off events local maxima of backward progression of the toe marker. We visually inspected the result of this automatic identification and applied manual corrections in the rare cases where events were misidentified. We have used this method of gait event detection successfully in the past and very rarely requires manual adjustment^30^.

All data between heel strikes were normalized to 100 time points. We subtracted the *baseline slow* mean from all data, including *baseline slow*, for every subject.

Throughout the manuscript and in the figures we refer to the individual feet by the speed with which they moved during the *split* condition. Specifically, the left foot was on the belt moving at 1.4 m s^−1^ during *split*, therefore we refer to this foot as *fast*. The right foot was on the belt moving at 0.7 m s^−1^ during *split*, so we refer to this foot as *slow*.

### Quantifying outcome variables

Analyzing the adaptation of our gait parameters on every step requires that each parameter is summarized by a single value. All of the data here are represented as a change in response from the average of the baseline *slow* steps ($\Delta$). We use the following definitions to quantify the use of the gait parameters on every step:

Spatial gait parameters

- Step Length: Distance from the trailing foot to the leading foot heel marker at the leading foot heel-strike in AP direction.
- Step Width: Distance from the trailing foot heel marker to the leading foot heel marker at the leading foot heel-strike in ML direction.

Temporal gait parameters

- Single Stance Time: Time between the swing foot pushoff and heel-strike.
- Double Stance Time Time between the stance foot heel-strike and the swing foot pushoff.

CoM referenced gait parameters (ML balance parameters)

- CoM: ML position of the middle of the posterior hip markers at heel strike of the swing foot.
- $\int$CoP-CoM: Displacement between the CoM and the CoP from the *slow* baseline steps, integrated over the single stance phase.
- Step-CoM: Displacement of the swing foot heel marker from the CoM at heel-strike.

We then quantified the symmetry between feet (*slow* and *fast*). Symmetry is defined as $\Delta$*fast* − $\Delta$*slow*. Following Finely et al.^7^, we quantified the rate of adaptation based on the number of steps it took for a variable to reach plateau. The plateau of the symmetry was defined as the average value during the last 50 steps, and the threshold for reaching plateau was defined as the step when the next 9 consecutive steps remained within 2 standard deviations of the plateau^7^. We refer to this value as the *number of steps to plateau*. We calculated the plateau for each participant and all gait parameters for the *split*. We also calculated the magnitude of symmetry change influenced by the perturbation (*split*) and aftereffect (*after*) which is defined as the magnitude of the asymmetry at the time of plateau.

### Statistical analysis

We investigate whether older adults adapt differently to a split belt treadmill paradigm compared to younger adults, with an emphasis on the ML control of balance. We ran statistics to test for two primary questions. (1) Do the gait parameters exhibit age differences in the plateau and magnitude of perturbation effects during the *split*, reflecting different strategies? (2) Do the gait parameters that are associated with the control of balance exhibit age differences in steps to plateau during the *split* condition and magnitude of aftereffects, compared to traditional parameters, reflecting separate neural control.

To test our hypotheses that aging affects how the gait parameters change throughout the split belt treadmill paradigm, we used lme4^31^ within R (version 3.5.1;^32^) to perform linear mixed effects analyses. We fit three linear mixed models to analyze how the gait parameters were modulated in the *split* and *after* conditions, and whether these modulations differed by age group and gait parameter. *Group* (younger vs older) and *gait parameters* were treated as fixed effects, and participant intercepts were random effects. Equation (1) provides the lmer model testing for age and gait parameter differences in steps to plateau during the split condition,

1 $$\begin{aligned} \texttt {Split\_Plateau} \sim \texttt {group} \cdot \texttt {gait\_parameters} + \texttt {1|Subject}, \end{aligned}$$

Equation (2) provides the lmer model testing for age and gait parameter differences in magnitude of plateau,

2 $$\begin{aligned} \texttt {Split\_Plateau\_Magnitude} \sim \texttt {group} \cdot \texttt {gait\_parameters} + \texttt {1|Subject}, \end{aligned}$$

and Eq. (3) provides the lmer model testing for gait parameter differences in magnitude of aftereffects (first 10 steps in *after* condition),

3 $$\begin{aligned} \texttt {After\_Magnitude} \sim \texttt {group} \cdot \texttt {gait\_parameters} + \texttt {1|Subject}. \end{aligned}$$

For each model we performed an ANOVA using Satterthwaite’s method^33^ implemented in the R-package lmerTest^34^ to obtain parameter-specific p values. We then performed posthoc pairwise analyses for each model by calculating the least squares means and estimating the 95% confidence intervals, using a Kenward–Roger approximation^35^ implemented in the R-package emmeans (version 1.4.1;^36^). We converted the $\Delta$ values into percent change from the average of the slow condition to enable pairwise-comparisons between gait parameters.

### Ethics approval and consent to participate

Subjects provided informed verbal and written consent to participate. Written informed consent was obtained from the individual for the publication of any potentially identifiable images or data included in this article. The experiment was approved by the University of Florida Institutional Review Board (IRB ID: IRB201801417). All methods were carried out in accordance with relevant guidelines and regulations.

### Consent for publication

All authors provided approval for publication.

## Results

All participants included in this analysis completed the treadmill walking trials without falling or tripping. In general, all participants adjusted multiple gait parameters to accommodate the different treadmill belt speeds.

**Figure 1:**
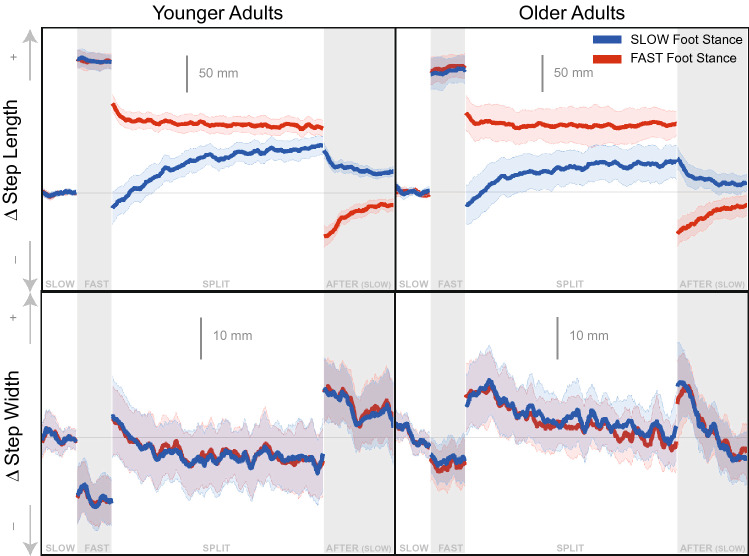
Spatial Gait Parameters adaptation time series graphs for younger (left column) and older (right column) adults. The time-series data were smoothed with a 2 step sliding window moving average for display purposes. The blue data corresponds to the right foot, and the foot that made contact with the *slow* belt during *split* condition. The red data corresponds to the left foot, and the foot that made contact with the *fast* belt during *split* condition. The bolded lines indicate the group mean. The shaded areas represent the 95% confidence interval of the group mean. All values are represented as a change from the average of the *slow* condition ($\Delta$). + indicates more forward for step length and wider for step width.

In Fig. 1 we observe step length increases from *slow* to *fast* baseline conditions. During *split*, step length is larger when the *fast* foot is in stance compared to the *slow* foot stance, but becomes more symmetric over time. During *after* the step length exhibits an aftereffect opposite to the modulation observed during *split*. Step width drops slightly from *slow* to *fast* baseline conditions. Step width increases initially in both the *split* and *after* conditions. Both groups generally show similar trends of adaptation throughout the *split* and *after* conditions.

**Figure 2:**
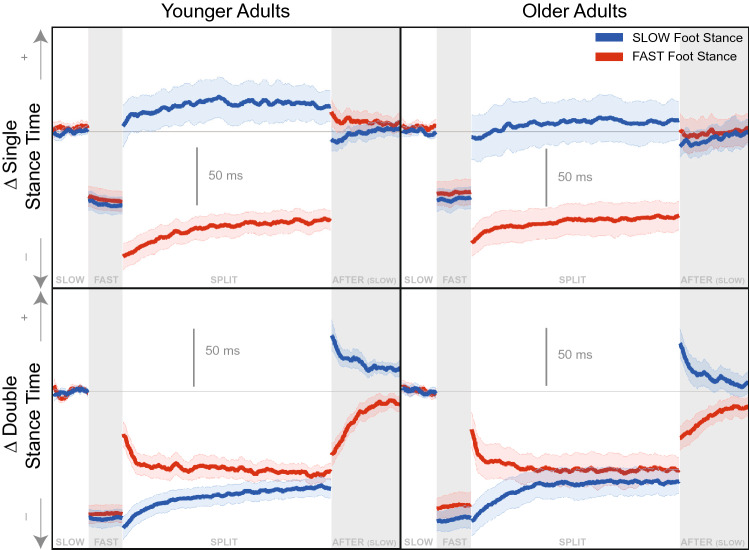
Temporal Gait Parameters adaptation time series graphs for younger (left column) and older (right column) adults. The time-series data were smoothed with a 2 step sliding window moving average for display purposes. The blue data corresponds to the right foot, and the foot that made contact with the *slow* belt during *split* condition. The red data corresponds to the left foot, and the foot that made contact with the *fast* belt during *split* condition. The bolded lines indicate the group mean. The shaded areas represent the 95% confidence interval of the group mean. All values are represented as a change from the average of the *slow* condition ($\Delta$). + indicates more time for both variables.

Figure 2 shows single and double stance time decreases from *slow* to *fast* baseline conditions. During the *split* condition, the two stance phases are modulated differently. The single stance time generally hovers around the stance time observed during the respective baseline condition (i.e. slow stance time during *split* was similar to that during *slow* baseline). The double stance time does not follow this same behavior. During *split*, the double stance time was longer when the fast stance foot was leading, but eventually the double stance phase becomes symmetric. During *after*, the double stance time exhibited similar aftereffects as step length, with a reversal in the difference between the two feet (i.e. double stance was longer when slow stance foot is leading).

**Figure 3:**
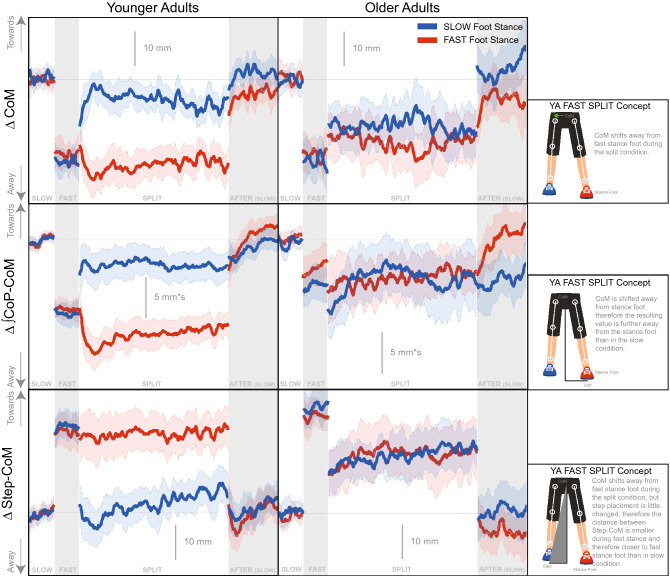
CoM Referenced Gait Parameters adaptation time series graphs for younger (left column) and older (right column) adults. The time-series data were smoothed with a 2 step sliding window moving average for display purposes. The blue data corresponds to the right foot, and the foot that made contact with the *slow* belt during *split* condition. The red data corresponds to the left foot, and the foot that made contact with the *fast* belt during *split* condition. The bolded lines indicate the group mean. The shaded areas represent the 95% confidence interval of the group mean. All values are represented as a change from the average of the *slow* condition ($\Delta$). Towards refers to in the direction of the stance foot.

Figure 3 depicts CoM referenced gait parameters that inform us about ML balance. We observed dramatic age differences in changes in the CoM variables in the *split* condition. During the baseline conditions, the two groups exhibit roughly similar behavior for these CoM referenced variables. The CoM shifted further away from the stance foot in the *fast* compared to the *slow* condition. Similarly, the $\int$CoP-CoM during the *fast* condition also shifted away from the stance foot relative to the *slow* condition. The step placement relative to the CoM shifted more towards the stance foot from *slow* to *fast* baseline conditions, mostly due to the shift of the CoM away from the stance foot. The behavior during the *split* condition is quite different for the younger compared to older adults. The younger adults quickly adopted an asymmetry in all three variables. During the *split* condition, the younger adults’ CoM referenced variables hover around the values that were observed during the respective baseline conditions for each stance foot. The older adults did not exhibit a similar, sustained asymmetry, during the *split* condition for the CoM referenced variables.

**Figure 4:**
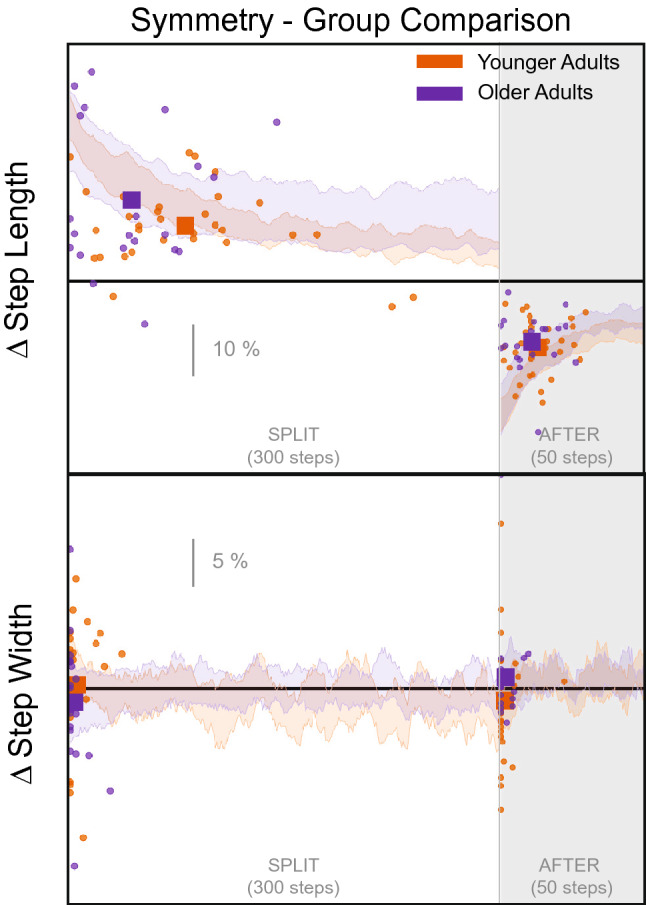
Spatial Gait Parameters Steps To Plateau graphs for younger (orange) and older (purple) adults for *split* and *after* conditions. The small circles indicate single subject steps to plateau and the larger squares indicate the group average. The shaded area represents the 95% confidence interval for the group average symmetry. The thick black horizontal line indicates perfect symmetry.

**Figure 5:**
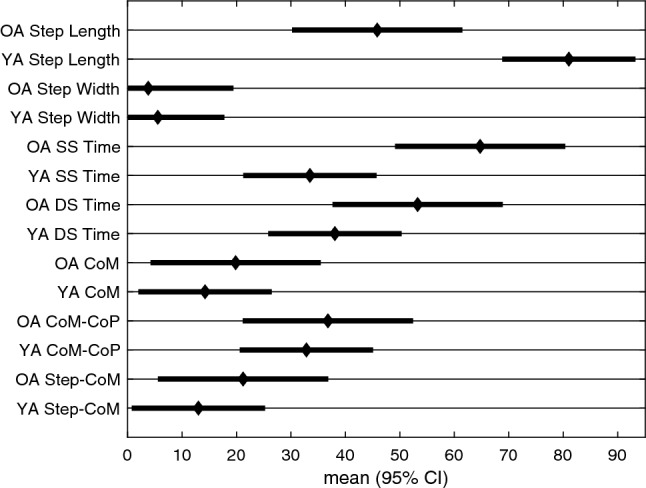
Split Steps to Plateau posthoc comparison figure: estimated 95% confidence intervals for % $\Delta$ steps to the plateau for the *split* condition [lmer Eq. (1)]. Confidence intervals that do not overlap reflects a significant difference between them.

**Figure 6:**
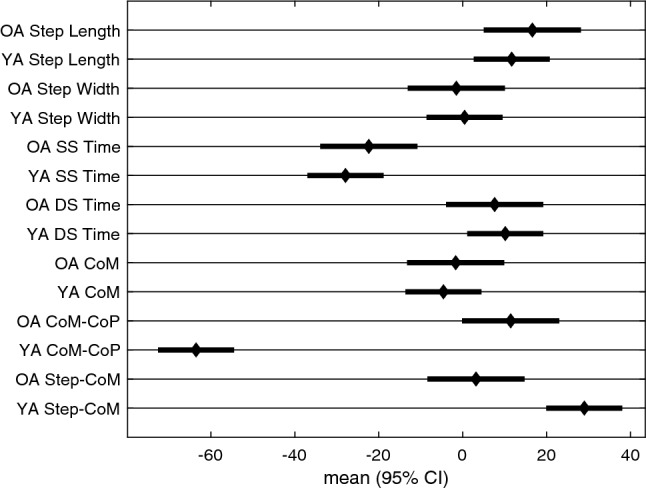
Split Magnitude of Plateau posthoc comparison figure: estimated 95% confidence intervals for % $\Delta$ magnitude of the plateau during the *split* condition [lmer Eq. (2)]. Confidence intervals that do not overlap reflects a significant difference between them.

Figure 4 displays the adaptation trajectories for the symmetry scores of the spatial gait parameters. The triangles indicate the individual steps to plateau (x-axis) and the associated magnitude (y-axis). The squares represent the group average. There was a large asymmetry in step length that reduces throughout the *split* condition and then reverses during the *after* condition, for both age groups. During the *split* condition, the step length rate of adaptation was significantly different between the younger and older adults, with younger adults taking longer to reach a plateau (95% confidence intervals do not overlap, Table 3, Fig. 5). The magnitude of plateau did not significantly differ between the two groups for step length (Table 5, Fig. 6). Step width did not exhibit significant asymmetry during *split* for either age group (Table 5, Fig. 6).

**Table 2:** Split Steps to Plateau ANOVA: results of the mixed model for the rate of adaptation of the gait parameters at the point at which plateau occurred for the condition.

Effect	Sum sq	Mean sq	NumDF	DenDF	F value	Pr(> F)
group	826	826	1	48	0.739	0.394
outcome_measure	1.25e+05	2.09e+04	6	288	18.7	<**0.001**
group:outcome_measure	2.89e+04	4.82e+03	6	288	4.32	<**0.001**

Bolded p-values indicate significance of the corresponding factor. The group factor refers to the younger and older adult categories and the outcome measure factor refers to the different gait parameters.

**Table 3:** Split Steps to Plateau Posthoc comparisons: estimated 95% confidence intervals for % $\Delta$ steps to the plateau for the *split* condition [lmer Eq. (1)].

Group	Outcome_measure	lsmean	SE	df	Lower. CL	Upper. CL
OA	Step length	45.8	7.95	327	30.2	61.5
YA	Step length	81	6.22	327	68.8	93.3
OA	Step width	3.79	7.95	327	-11.8	19.4
YA	Step width	5.55	6.22	327	-6.69	17.8
OA	SS time	64.7	7.95	327	49.1	80.4
YA	SS time	33.5	6.22	327	21.2	45.7
OA	DS time	53.3	7.95	327	37.6	68.9
YA	DS time	38.1	6.22	327	25.8	50.3
OA	CoM	19.8	7.95	327	4.21	35.5
YA	CoM	14.2	6.22	327	1.99	26.5
OA	CoM-CoP	36.8	7.95	327	21.2	52.4
YA	CoM-CoP	32.8	6.22	327	20.6	45.1
OA	Step-CoM	21.2	7.95	327	5.58	36.8
YA	Step-CoM	13	6.22	327	0.763	25.2

Confidence intervals that do not overlap reflects a significant difference between them.

**Table 4:** Split Magnitude of Plateau ANOVA: results of the mixed model for the magnitude of asymmetry of the gait parameters at the point at which plateau occurred for the split [lmer Eq. (2)].

Effect	Sum sq	Mean sq	NumDF	DenDF	F value	Pr(> F)
Group	5.67e+03	5.67e+03	1	336	8.63	**0.0035**
Outcome_measure	8.6e+04	1.43e+04	6	336	21.8	<**0.001**
Group:outcome_measure	6.93e+04	1.15e+04	6	336	17.6	<**0.001**

Bolded p-values indicate significance of the corresponding factor. The group factor refers to the younger and older adult categories and the outcome measure factor refers to the different gait parameters.

**Table 5:** Split Magnitude of Plateau posthoc comparisons: estimated 95% confidence intervals for % $\Delta$ magnitude of the plateau for the *split* condition [lmer Eq. (2)].

Group	Outcome_measure	lsmean	SE	df	Lower. CL	Upper. CL
OA	Step length	16.6	5.88	336	5.04	28.2
YA	Step length	11.7	4.61	336	2.63	20.8
OA	Step width	− 1.51	5.88	336	− 13.1	10.1
YA	Step width	0.457	4.61	336	− 8.6	9.52
OA	SS time	− 22.4	5.88	336	− 33.9	− 10.8
YA	SS time	− 27.9	4.61	336	− 37	− 18.9
OA	DS time	7.62	5.88	336	− 3.95	19.2
YA	DS time	10.2	4.61	336	1.1	19.2
OA	CoM	− 1.66	5.88	336	− 13.2	9.91
YA	CoM	− 4.57	4.61	336	− 13.6	4.49
OA	CoM-CoP	11.4	5.88	336	− 0.129	23
YA	CoM-CoP	− 63.5	4.61	336	− 72.6	− 54.5
OA	Step-CoM	3.18	5.88	336	− 8.39	14.8
YA	Step-CoM	29	4.61	336	19.9	38.1

**Figure 7:**
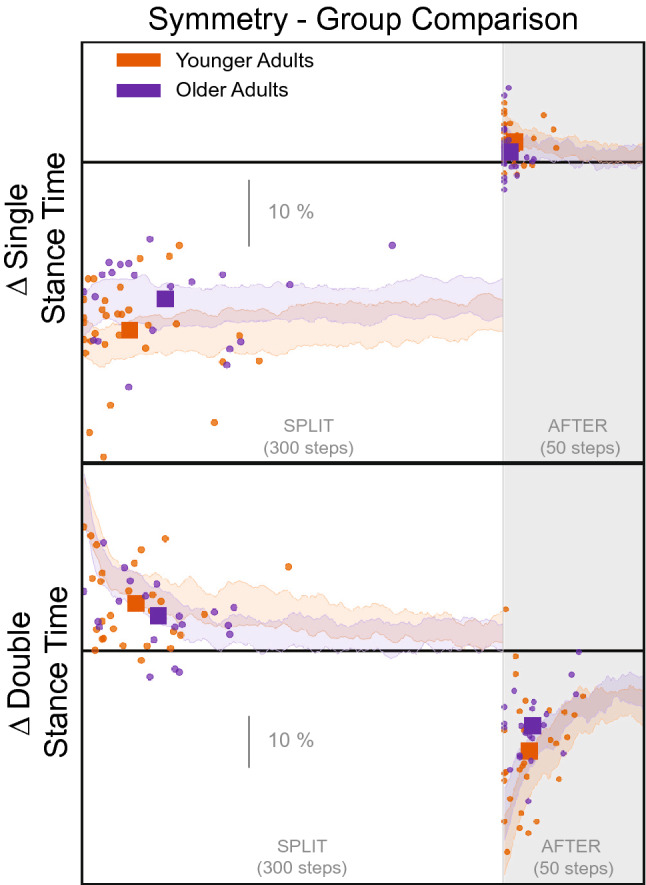
Temporal Gait parameters steps to plateau graphs for younger (orange) and older (purple) adults for *split* and *after* conditions. The small circles indicate single subject steps to plateau and the larger squares indicate the group average. The shaded area represents the 95% confidence interval for the group average symmetry. The thick black horizontal line indicates perfect symmetry.

Figure 7 displays the adaptation trajectories for the symmetry scores of the temporal gait parameters. There was a rather constant asymmetry for single stance time for both groups during the *split* condition. The single stance time rate of adaptation differed between the groups, with older adults taking longer to reach a plateau (95% CIs do not overlap, Table 3, Fig. 5). Double stance time exhibits a similar asymmetry to that observed in step length, with an eventual convergence to symmetry during the *split* condition. Neither steps to plateau nor the magnitude of plateau were significantly different between age groups for double stance time (Tables 3, 5, Figs. 5, 6).

**Figure 8:**
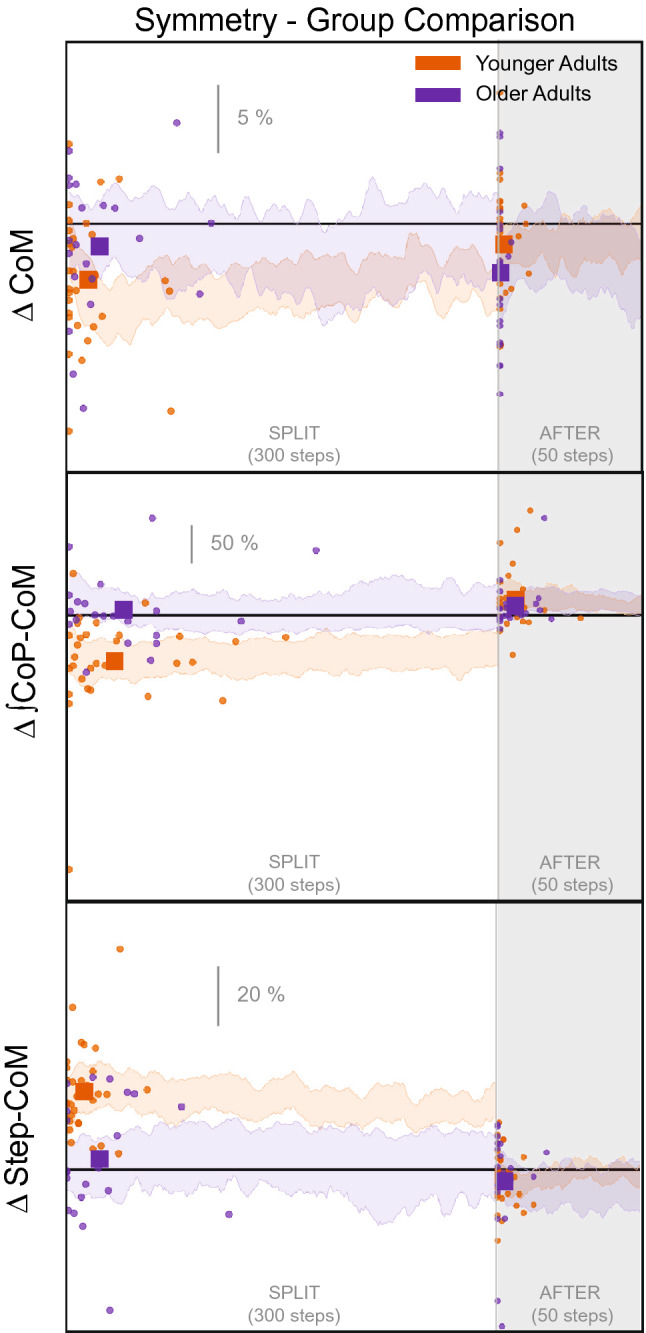
CoM referenced Gait parameters steps to plateau graphs for younger (orange) and older (purple) adults for *split* and *after* conditions. The small circles indicate single subject steps to plateau and the larger squares indicate the group average. The shaded area represents the 95% confidence interval for the group average symmetry. The thick black horizontal line indicates perfect symmetry.

Figure 8 displays the adaptation trajectories for the symmetry scores of the CoM gait parameters. We observed significant asymmetry in the CoM referenced variables, at least for the younger adults, during the *split* condition. The rate of adaptation during *split* is not significantly different between groups for any CoM referenced variables (Tables 2, 3, Fig. 5). However, there was a significant difference between groups for the magnitude of plateau for Step-CoM and CoM-CoP, where the younger adults had greater asymmetry in both of these parameters (95% CIs do not overlap, Tables 4, 5, Fig. 6).

**Figure 9:**
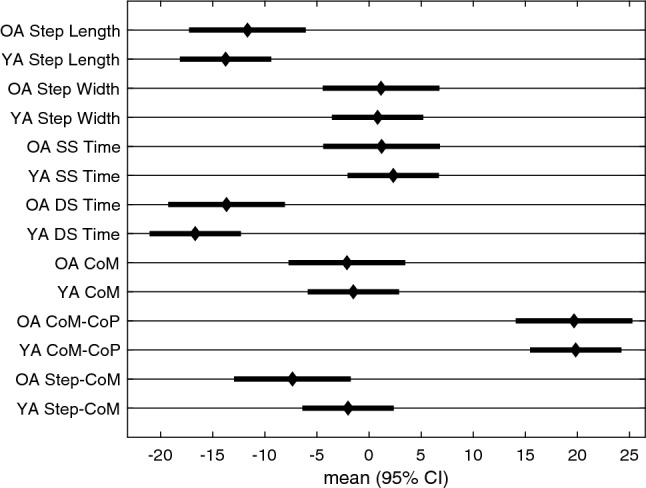
After Magnitude Posthoc comparison figure: estimated 95% confidence intervals for % $\Delta$ magnitude within the first ten steps of the *after* condition [lmer Eq. (3)]. Confidence intervals that do not overlap reflects a significant difference between them.

Tables 6, 7, and Fig. 9 display the ANOVA and Posthoc comparison results for testing the differences in magnitude of aftereffects (% $\Delta$ in first 10 steps of *after* condition). Step length, double stance time, CoM-CoP are the only variables that had significant aftereffects for both groups, and Step-CoM showed an aftereffect for the older adults (95% CI did not overlap with zero). Relative to the change observed during *split*, the aftereffects were in the opposite direction for all of these variables, with the exception of CoM-CoP for the older adults.

**Table 6:** After Magnitude ANOVA: results of the mixed model for the magnitude of asymmetry of the gait parameters at the beginning of the after condition [lmer Eq. (3)]. Bolded p-values indicate significance of the corresponding factor.

Effect	Sum sq	Mean sq	NumDF	DenDF	F value	Pr(> F)
Group	5.46	5.46	1	336	0.0355	0.851
Outcome_measure	3.73e+04	6.21e+03	6	336	40.4	<**0.001**
Group:outcome_measure	508	84.7	6	336	0.551	0.769

The group factor refers to the younger and older adult categories and the outcome measure factor refers to the different gait parameters.

**Table 7:** After Magnitude posthoc comparisons: estimated 95% confidence intervals for % $\Delta$ magnitude within the first ten steps of the *after* condition (lmer equation 3).

Group	Outcome_measure	lsmean	SE	df	Lower. CL	Upper. CL
OA	Step Length	− 11.7	2.85	336	− 17.3	− 6.07
YA	Step Length	− 13.8	2.23	336	− 18.1	− 9.38
OA	Step width	1.15	2.85	336	− 4.45	6.74
YA	Step width	0.821	2.23	336	− 3.56	5.2
OA	SS time	1.21	2.85	336	− 4.39	6.8
YA	SS time	2.33	2.23	336	− 2.05	6.71
OA	DS time	− 13.7	2.85	336	− 19.3	− 8.08
YA	DS time	− 16.7	2.23	336	− 21.1	− 12.3
OA	CoM	− 2.12	2.85	336	− 7.72	3.48
YA	CoM	− 1.51	2.23	336	− 5.89	2.87
OA	CoM-CoP	19.7	2.85	336	14.1	25.3
YA	CoM-CoP	19.8	2.23	336	15.4	24.2
OA	Step-CoM	− 7.35	2.85	336	− 12.9	− 1.75
YA	Step-CoM	− 2.02	2.23	336	− 6.4	nn2.37

Confidence intervals that do not overlap reflects a significant difference between them.

## Discussion

Our goal was to analyze multiple gait parameters that span both the AP and ML directions during split belt treadmill adaptation in young and older adults to determine whether they adapt differently. Previous split belt treadmill studies have shown that older adults generally adapt less, more slowly, and show reduced aftereffects relative to younger adults^28,37,38^. Many of these studies have not included ML gait parameters in their analysis, though. Examining ML balance control is necessary to gain a comprehensive understanding of how humans adapt to the split belt treadmill, and how this process differs by age.

Here we quantified multiple gait parameters that have been used to study balance during walking. Figs. 1, 2, and 3 reveal that most of the gait parameters are altered throughout the split belt treadmill protocol, including those that are thought to contribute to ML balance. The most commonly analyzed gait parameters (step length and stance times) show clear effects of the split belt treadmill protocol, including aftereffects and group differences. However, we found that the older adults reached the plateau for their step length more quickly than younger adults. We also see some notable changes in the other parameters, including an initial modification of step width. Step width increases slightly during the *split* and *after* conditions in both age groups, likely to increase stability. The CoM referenced variables exhibit the largest difference between groups in regards to magnitude of the perturbation (*split* condition).

The CoM referenced variables provide information about how balance is being controlled. Specifically, the younger adults quickly adopt an asymmetry in their control of balance. This asymmetry is observed as a shift of the average body center away from the *fast* foot, which spends less time in stance, towards the *slow* foot, which spends more time in stance. This finding of asymmetry in an ML balance parameter in young adults is comparable to reports of margin of stability asymmetry from Buurke et al.^15^. The older adults, in contrast, maintain symmetry between their body center and the average foot placement on both sides. This frontal-plane asymmetry adopted by the younger adults is beneficial due to utilization of the gravitational forces acting on the body. The gravitational force accelerates the body mass with a lever arm given by the displacement of the center of mass (CoM) from the CoP^20^. This gravitational acceleration creates the rhythmic lateral oscillation of the CoM with the gait cycle, by always accelerating the CoM away from the current stance foot. Walking on a split-belt treadmill induces an asymmetry between the stance times for each foot. With an otherwise unchanged gait pattern, such a difference in stance times would lead to a difference in total acceleration between the two sides, because the gravitational force has less time to pull the body away from the fast foot compared to the slow foot. Integrated over time, such a difference in gravitational acceleration would result in a sideways movement of the whole body, away from the center of the treadmill. To stay in the center of the treadmill, this difference in gravitational acceleration must be compensated. One way to compensate is to change the lever arm of the gravitational force, making it larger for the fast foot and smaller for the slow foot by shifting the body center towards the *slow* leg. The larger lever arm from this shift cancels out the shorter time over which the gravitational force is applied during the *fast* stance period, resulting in equal total acceleration in both directions from the gravitational force. We suggest that this is the solution adopted, on average, by the young participants.

The older participants, in contrast, do not use this strategy to compensate for the difference in gravitational acceleration due to asymmetric stance time. Another way to generate lateral acceleration is to actively push against the ground to move the body back towards the *slow* leg in order to keep it in the center of the treadmill. Such an active muscle force would most likely be generated using the ankle roll mechanism, shifting the CoP in the direction of the *fast* leg^39,40^. Using muscle force to generate these corrections requires substantial metabolic energy, enough to move the whole body mass. Shifting the foot placement to passively exploit gravitational forces, in comparison, requires minimal metabolic energy, since only the mass of the leg is moved by active muscle forces. We suggest that this solution of actively moving the whole body is adopted, on average, by the older participants in our study, even though it is metabolically more expensive.

**Figure 10:**
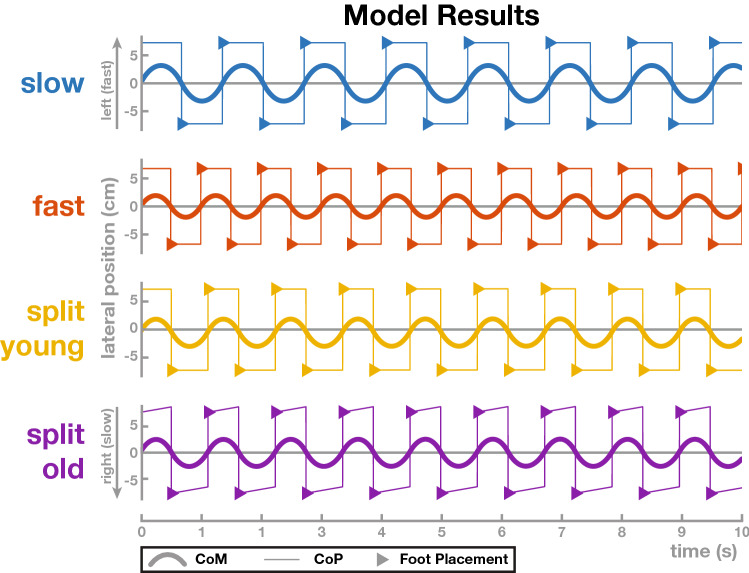
Single-link Inverted Pendulum Model: Results of the linearized single-link inverted pendulum models in the frontal plane. For each model, the thick solid line shows the CoM, the thin line the CoP and the triangles the foot placement for each step. The medium grey line indicates the mid-line between the left and right foot placements. The top two models show tied-belt walking in the slow (0.7 m s^−1^, blue) and fast (1.4 m s^−1^, red) baseline conditions. The bottom two models show split-belt walking with two different control approaches to stabilize the medial-lateral position on the treadmill. The “split young” model (yellow) uses asymmetric foot placement control, resulting in a systematic shift of the CoM relative to the mid-line between the feet. The “split old” model (purple) uses CoP modulation to shift the CoM to the left throughout each step, resulting in symmetric foot placement, but likely requiring active muscle forces.

To elucidate the different coordination patterns between the step location and the center of mass in the medial-lateral direction, we adapted a previously developed model of walking control in the frontal plane to include split-belt walking. We provide an overview of the model here and refer the reader to^41^ for details. The model consists of a single-link inverted pendulum with a mass-less leg that is instantaneously switched to a new contact point when a step is taken after a fixed time^20^. We used this model to reproduce the coordination pattern between body center and foot placement in the frontal plane observed in split-belt walking in the younger and older adult groups. We used the base model with only foot placement control to generate walking trajectories similar to tied-belt walking in both the *slow* and *fast* speed baseline conditions. We set the step duration to the experimentally observed average values of 0.68 s in the *slow* and 0.50 s in the *fast* condition and used constrained gradient descent optimization implemented in the MATLAB function fmincon to find parameters that generate the experimentally observed average step width of 0.145 m in the *slow* and 0.135 m in the *fast* condition. The optimization successfully found constant offset parameters *b* = 0.013 m in the *slow* and *b* = 0.021 m in the *fast* condition. The resulting trajectories are shown in Figure 10, with *slow* in blue and *fast* in red. To model split-belt walking, we set the step duration to the experimentally observed averages of 0.51 s for *slow* steps and 0.61 s for *fast* steps. We added an additional constraint to the optimization, requiring that the average lateral position remains constant over time, i.e. that the body stays in the center of the treadmill, and used the average step width of 0.145 m as the optimization goal. For the younger adults, we used separate offset control parameters for each leg. The optimization successfully found parameters $b_\text{fast}$ = 0.023 m and $b_\text{slow}$ = 0.015 m. The resulting trajectories, shown in orange in Fig. 10, show that the CoM is shifted away from the *fast*, left leg and towards the *slow*, right leg, reproducing the asymmetry observed in the *young* participants. For the older adults, we used a single constant offset parameter, resulting in symmetric foot placement relative to the CoM, as in tied-belt walking. The optimization was not successful in this case, confirming that additional force is required to compensate for the asymmetric gravitational acceleration, as described above. We then added a compensating force in the form of a constant-rate CoP shift. With this additional mode of lateral control, the optimization successfully found parameters for constant offset $b = 0.019$ m and CoP shift rate $c = 0.019$ m s^−1^. The resulting trajectories, shown in purple in Fig. 10, show symmetric foot placement relative to the CoM, as observed in the older adults.

The model results support our hypothesis that the asymmetry adopted by the younger adults is beneficial by limiting the amount of active forces applied to maintain the CoM within the base of support. Given that the CoM acceleration is proportional to the distance between the CoP and the CoM^20^, the shortened stance time decreases the amount of time for gravitational forces to act on the CoM. The younger adults appear to shift the CoM to one side in order to create the same total absolute acceleration under both feet. This potentially comes at a cost of stability, decreasing the margin of stability on one side, but may contribute to enhanced metabolic efficiency. The older adults may sacrifice metabolic efficiency in order to maintain symmetry in their balance parameters. Recent work has investigated whether asymmetry in the AP direction is metabolically inefficient^13,42^, with mounting evidence suggesting asymmetry is more efficient when one leg is constrained^43^, but to our knowledge metabolic cost of asymmetry in the ML direction has not been studied. However, it is conceivable that the current findings are in line with recent work suggesting step time asymmetry is optimized to reduce metabolic expenditure^44^. Future work will be needed in order to determine whether the lack of asymmetry in the ML direction is metabolically inefficient for older adults, and which specific muscles are being used to counteract the constraints. A hip strategy may be used to keep the older adults balance parameters more symmetric, which would also lead to increased metabolic demands^45^. We believe the current results suggest the younger adults are more able to fine-tune their body configuration compared to the older adult group to achieve the same solution with less effort.

The magnitude and direction of aftereffects in the *after* condition indicates whether a gait parameter change was a temporary, strategic adjustment or a longer adaptation. That is, the presence of aftereffects is typically interpreted as reflecting adaptive processes, whereas a lack of aftereffects suggests that a change in performance in response to a perturbation was more of a transient strategy^46^. The carryover and opposite effect of motor adaptations into the *after* condition is the hallmark sign of sensorimotor adaptation^47,48^. Here we use the magnitude of aftereffects to confirm whether the gait parameter changes during *split* are adaptive changes or instead reflect transient strategies. Four gait parameters showed significant aftereffects, step length, double stance time, $\int$CoP-CoM, and Step-CoM. The step width increase is not detected by our symmetry measure because it increases symmetrically. Nevertheless, step width increases in both *split* and *after* suggest a quick strategic effect to deal with the split belt treadmill paradigm. It is often thought that increasing step width increases stability^40,49^, so increasing step width at the beginning of each perturbation condition (*split* and *after*) will increase stability enabling focus on the actual problem (feet moving at different speeds while in stance phase). On the other hand, step length and double stance time show significant aftereffects in the opposite direction of that observed during *split* for both age groups. The Step-CoM and $\int$CoP-CoM show aftereffects in the opposite direction only for the younger adults. This suggests a motor adaption occurred during *split*, and required readjustment when the belts were again moving at the same speed (*after*). Further, the rate of adaptation (steps to plateau) may be informative for determining the type of strategy employed. Step width adapts rather quickly, relative to step length and double stance time, for both groups. Therefore, we suggest that a shorter plateau reflects a strategic response for that gait parameter. However, the CoM referenced variables showed unique patterns. For example, the step-CoM showed a large asymmetry during the *split* condition, but relatively quick adaptation, followed by an aftereffect in the opposite direction of that shown in *split*. Therefore, it is not clear what learning procedure may be governing this balance control parameter.

During the *split* condition, there was a significant difference between groups for step length and single stance time. The younger adults took more steps to reach a plateau than the older adults and younger adults also adapted step length toward symmetry to a greater extent compared to older adults. The younger adults adapted faster in single stance time. Previous split belt treadmill studies have shown that older adults generally adapt less, more slowly, and show reduced aftereffects relative to younger adults^28,37,38^. However, we believe this finding is influenced by the fact that the younger adults appear to make slow adaptations to their step length throughout the *split* condition, and even seem to continue past the displayed 300 steps (Fig. 4). More importantly, the current results suggest that the largest difference between younger and older adults is in the ML balance parameters. These differences are observed in the group average, but it is evident that some older adults use similar strategies as younger adults as observed in Figs. 4, 7, 8. The AP parameters may only become compromised once the balance parameters are severely altered or the task requires additional neural resources. Sombric et al found that poorer cognitive task switching performance was associated with difficulty switching between split belt treadmill and overground walking, suggesting that age declines in cognitive performance may interact with split belt treadmill adaptation^38,50^. We hypothesize that this may be the case for individuals with pronounced mobility difficulties or neurological populations that struggle with balance (e.g.,  stroke or Parkinson’s Disease) or show more pronounced cognitive declines (Alzheimer’s).

### Limitations

There are several limitations to this work. We acknowledge that the finding of older adults adapting their step length quicker than younger adults may be a limitation of the analysis methods. As younger adults appear to continue adaptation beyond the observed steps in Fig. 1, the older adults, on average, do not adapt to the same degree as the younger adults. We also acknowledge that the decision to fix the speed of the belt to 1.4 m s^−1^ for fast and .7 m/s for slow, for all participants, may have been more perturbing for older adults that generally walk slower than the prescribed fast speed. However, age-related differences in split-belt treadmill adaptation has been demonstrated in both studies using fixed speeds (e.g.,^7,13,51–54^) and preferred-walking speeds (e.g.^28^). Furthermore, the decision to fix the fast belt to the left foot and slow foot to the right may have influenced participant strategy, but since we were mostly concerned with symmetry scores the effects of individual feet should matter less. Additionally, it has been reported previously that the ankle roll mechanism is the likely major contributor to the CoP modulation^55^, however it is possible torque produced by the hips or arms^56^, foot yaw rotation^57^, or a modulation of the push-off^58^ can contribute to the relationship between ground reaction forces and the CoM. Reommich et al and Ogawa et al reported adaptation of the AP ground reaction forces which may be indicative of a push-off modulation^59,60^. We do not know the extent to which stance limb push-off propulsion contributes to ML body movement, or the extent to which this could be a contributing factor in the modulation of the CoM-CoP and other gait parameters in the current study. Additionally, for the safety of our participants we installed handrails on each side of the treadmill for this experiment. We explicitly instructed participants to hold on to the handrails as the treadmill started, for each condition, then let go once comfortable. This may have led to diminished effects, particularly in the ML direction. Although participants were not actively using the handrail, knowledge of the handrail location may have limited their ML movement.

### Future directions

We performed a comprehensive analysis of this data set, but there are still many unknowns that can be uncovered with more extensive data acquisitions in the future. Specifically, future work should include a detailed analysis of joint movements and associated muscle activity in order to determine how the younger and older adults’ control of balance is affected by the split belt treadmill paradigm. Electromyography will provide information about whether a movement is actively or passively generated. The addition of electromyography would verify our suggestion that the younger adults medial-lateral asymmetry is indeed passive, or rather that the older adults lack of asymmetry is actively generated. Furthermore, full body kinematics are critical for unraveling the details of locomotor behavior. For example, our modeling work suggests that the older adults must generate some force to overcome the medial-lateral asymmetry induced by the *split* condition, but we cannot determine whether these forces are generated by hip or trunk torques without full body kinematics. Additionally, the neural correlates of the different gait parameters may provide more understanding about why some gait parameters adapt more quickly or do not show any aftereffects. Therefore, future studies should consider acquiring brain activity data to gain a more comprehensive understanding of the neural controls of locomotor adaptation.

### Conclusion

We investigated whether younger and older adults adapted their gait parameters differently during a split belt treadmill walking paradigm. Specifically, we analyzed unique gait parameters that have not been previously analyzed in a split belt treadmill walking paradigm including control of balance parameters. The goal was to determine whether ML parameters are changed during split belt treadmill adaptation walking in an age-dependent fashion. The current findings support the idea that participants alter ML balance control during split belt treadmill walking in a manner that differs by age. Step Width is modulated initially by both younger and older adults, possibly to increase stability to allow for neural resources to focus on the actual problem of the feet moving at different speeds during stance. The age differences in the CoM referenced variables between groups suggest more fine-tuned control of this ML gait parameter for the younger adults, possibly enhancing metabolic efficiency. Further, our modeling work supports that asymmetry during the *split* condition may be intentional in order to exploit passive dynamics. Our work suggests that ML balance control must be actively modulated, even in the presence of AP perturbations. Moreover, younger and older adults approach this problem differently. Younger adults adopt a process that may be more metabolically efficient, whereas older adults adopt a process that maintains gait symmetry.

## Data Availability

Data will be made available upon request. Data processing code will be made available upon request. Modeling code is available on github (https://github.com/hendrikreimann/SplitBeltWalker).

## References

[1] Pigman J, Reisman DS, Pohlig RT, Wright TR, Crenshaw JR (2019). The development and feasibility of treadmill-induced fall recovery training applied to individuals with chronic stroke. BMC Neurol..

[2] Allin LJ (2020). Perturbation-based balance training targeting both slip- and trip-induced falls among older adults: A randomized controlled trial. BMC Geriatr..

[3] Sawers A, Ting LH (2015). Beam walking can detect differences in walking balance proficiency across a range of sensorimotor abilities. Gait Posture.

[4] Gates DH, Scott SJ, Wilken JM, Dingwell JB (2013). Frontal plane dynamic margins of stability in individuals with and without transtibial amputation walking on a loose rock surface. Gait Posture.

[5] Reimann H, Ramadan R, Fettrow T, Hafer JF, Jeka J (2020). Interactions between different age-related factors affecting balance control in walking. Front. Sports Active Living.

[6] Vandevoorde K, de Xivry JJ (2019). Internal model recalibration does not deteriorate with age while motor adaptation does. Neurobiol. Aging.

[7] Finley JM, Long A, Bastian AJ, Torres-Oviedo G (2015). Spatial and temporal control contribute to step length asymmetry during split-belt adaptation and hemiparetic gait. Neurorehabil. Neural Repair.

[8] Prokop T, Berger W, Zijlstra W, Dietz V (1995). Adaptational and learning processes during human split-belt locomotion: Interaction between central mechanisms and afferent input. Exp. Brain Res..

[9] Finley JM, Statton MA, Bastian AJ (2014). A novel optic flow pattern speeds split-belt locomotor adaptation. J. Neurophysiol..

[10] Sato S, Choi JT (2021). Neural control of human locomotor adaptation: Lessons about changes with aging. Neuroscientist.

[11] Reisman DS, Block HJ, Bastian AJ (2005). Interlimb coordination during locomotion: What can be adapted and stored?. J. Neurophysiol..

[12] Finley JM, Bastian AJ, Gottschall JS (2013). Learning to be economical: The energy cost of walking tracks motor adaptation. J. Physiol..

[13] Sánchez N, Simha SN, Donelan JM, Finley JM (2018). Taking advantage of external mechanical work to reduce metabolic cost: The mechanics and energetics of split-belt treadmill walking. Physiol. Behav..

[14] Roper JA, Roemmich RT, Tillman MD, Terza MJ, Hass CJ (2017). Split-belt treadmill walking alters lower extremity frontal plane mechanics. J. Appl. Biomech..

[15] Buurke TJW, Lamoth CJC, Vervoort D, Woude LHVVD, Otter RD (2018). Adaptive Control of Dynamic Balance in Human Gait on a Split-belt Treadmill.

[16] Vervoort D (2020). Adaptive control of dynamic balance across the adult lifespan. Med. Sci. Sports Exerc..

[17] Bauby CE, Kuo AD (2000). Active control of lateral balance in human walking. J. Biomech..

[18] O’Connor SM, Kuo AD (2009). Direction-dependent control of balance during walking and standing. J. Neurophysiol..

[19] Collins SH, Kuo AD (2013). Two independent contributions to step variability during over-ground human walking. PLoS ONE.

[20] Hof AL, Gazendam MGJ, Sinke WE (2005). The condition for dynamic stability. J. Biomech..

[21] Buurke TJ, Lamoth CJ, van der Woude LH, Hof AL, den Otter R (2019). Bilateral temporal control determines mediolateral margins of stability in symmetric and asymmetric human walking. Sci. Rep..

[22] Hupfeld KE (2020). In vivo brain glutathione is higher in older age and correlates with mobility. BioRxiv.

[23] Rossi S, Hallett M, Rossini PM, Pascual-Leone A, Safety of TMS Consensus Group (2009). Safety, ethical considerations, and application guidelines for the use of transcranial magnetic stimulation in clinical practice and research. Clin. Neurophysiol..

[24] De Jager CA, Budge MM, Clarke R (2003). Utility of TICS-M for the assessment of cognitive function in older adults. Int. J. Geriatr. Psychiatry.

[25] Nasreddine ZS (2005). The Montreal Cognitive Assessment, MoCA: A brief screening tool for mild cognitive impairment. J. Am. Geriatr. Soc..

[26] Carson N, Leach L, Murphy KJ (2018). A re-examination of Montreal Cognitive Assessment (MoCA) cutoff scores. Int. J. Geriatr. Psychiatry.

[27] Kadaba M, Ramakrishnan H, Wooten M (1990). Measurement of lower extremity kinematics during level walking. J. Ortho.

[28] Roemmich RT (2014). Locomotor adaptation and locomotor adaptive learning in Parkinsons disease and normal aging. Clin. Neurophysiol..

[29] Yang F, Pai Y-C (2014). Can sacral marker approximate center of mass during gait and slip-fall recovery among community-dwelling older adults?. J. Biomech..

[30] Fettrow T, Dibianca S, Vanderlinde F (2020). Flexible recruitment of balance mechanisms to environmental constraints during walking. Front. Virtual Reality.

[31] Bates, D., Mächler, M., Bolker, B. M. & Walker, S. C. Fitting linear mixed-effects models using lme4. *Journal of Statistical Software* **67** (2015). arXiv:1406.5823.

[32] R Core Team. *R: A Language and Environment for Statistical Computing*. (R Foundation for Statistical Computing, 2018). https://www.R-project.org/.

[33] Fai AH-T, Cornelius PL (1996). Approximate F-tests of multiple degree of freedom hypotheses in generalized least squares analyses of unbalanced split-plot experiments. J. Stat. Comput. Simul..

[34] Kuznetsova A (2017). lmerTest package: Tests in linear mixed effects models. J. Stat. Softw..

[35] Halekoh U, Højsgaard S (2015). A Kenward–Roger approximation and parametric bootstrap methods for tests in linear mixed models: The R package pbkrtest. J. Stat. Softw..

[36] Lenth RV (2016). Least-squares means: The R package lsmeans. J. Stat. Softw..

[37] Bruijn SM, Van Impe A, Duysens J, Swinnen SP (2012). Split-belt walking: Adaptation differences between young and older adults. J. Neurophysiol..

[38] Sombric CJ, Harker HM, Sparto PJ, Torres-Oviedo G (2017). Explicit action switching interferes with the context-specificity of motor memories in older adults. Front. Aging Neurosci..

[39] Hof AL, Duysens J (2018). Responses of human ankle muscles to mediolateral balance perturbations during walking. Hum. Movement Sci..

[40] Reimann H, Fettrow T, Jeka J (2018). Strategies for the control of balance during locomotion. Kinesiol. Rev..

[41] Reimann H (2017). Complementary mechanisms for upright balance during walking. PLoS ONE.

[42] Sánchez N, Park S, Finley JM (2017). Evidence of energetic optimization during adaptation differs for metabolic, mechanical, and perceptual estimates of energetic cost. Sci. Rep..

[43] Browne MG, Smock CS, Roemmich RT (2021). The human preference for symmetric walking often disappears when one leg is constrained. Sci. Rep..

[44] Stenum J, Choi JT (2020). Step time asymmetry but not step length asymmetry is adapted to optimize energy cost of split-belt treadmill walking. J. Physiol..

[45] Pieper NL (2021). The metabolic and mechanical consequences of altered propulsive force generation in walking. J. Biomech..

[46] Taylor JA, Ivry RB (2012). The role of strategies in motor learning. Ann. N. Y. Acad. Sci..

[47] Martin Ta, Keating JG, Goodkin HP, Bastian aJ, Thach WT (1996). Throwing while looking through prisms: I. Focal olivocerebellar lesions impair adaptation. Brain.

[48] Fernández-Ruiz J, Díaz R (1999). Prism adaptation and aftereffect: Specifying the properties of a procedural memory system. Learn. Mem..

[49] Bruijn SM, Van Dieën JH (2018). Control of human gait stability through foot placement. J. R. Soc. Interface.

[50] Sombric CJ, Torres-Oviedo G (2020). Augmenting propulsion demands during split-belt walking increases locomotor adaptation of asymmetric step lengths. J. NeuroEng. Rehabil..

[51] Morton SM, Bastian AJ (2006). Cerebellar contributions to locomotor adaptations during splitbelt treadmill walking. J. Neurosci..

[52] Jayaram G (2012). Modulating locomotor adaptation with cerebellar stimulation. J. Neurophysiol..

[53] Hoogkamer W (2015). Adaptation and aftereffects of split-belt walking in cerebellar lesion patients. J. Neurophysiol..

[54] Malone LA, Bastian AJ (2016). Age-related forgetting in locomotor adaptation. Neurobiol. Learn. Mem..

[55] Fettrow TD (2019). Interdependence of balance mechanisms during walking. PLoS ONE.

[56] Otten E (1999). Balancing on a narrow ridge: Biomechanics and control. Philos. Trans. R. Soc. B.

[57] Rebula JR, Ojeda LV, Adamczyk PG, Kuo AD (2017). The stabilizing properties of foot yaw in human walking. J. Biomech..

[58] Kim M, Collins SH (2015). Once-per-step control of ankle-foot prosthesis push-off work reduces effort associated with balance during walking. J. NeuroEng. Rehabil..

[59] Roemmich R, Stegemöller E, Hass C (2012). Lower extremity sagittal joint moment production during split-belt treadmill walking. J. Biomech..

[60] Ogawa T, Kawashima N, Ogata T, Nakazawa K (2014). Predictive control of ankle stiffness at heel contact is a key element of locomotor adaptation during split-belt treadmill walking in humans. J. Neurophysiol..

